# Correlation between serum levels of proprotein convertase subtilisin/kexin type 9 (PCSK9) and atherogenic lipoproteins in patients with coronary artery disease

**DOI:** 10.1186/s12944-016-0339-8

**Published:** 2016-09-22

**Authors:** Tsuyoshi Nozue, Hiroaki Hattori, Kazuyuki Ogawa, Takeshi Kujiraoka, Tadao Iwasaki, Tsutomu Hirano, Ichiro Michishita

**Affiliations:** 1 Division of Cardiology, Department of Internal Medicine, Yokohama Sakae Kyosai Hospital, Federation of National Public Service Personnel Mutual Associations, 132 Katsura-cho, Sakae-ku, Yokohama, 247-8581 Japan; 2 Advanced Medical Technology and Development Division, BML Inc., Kawagoe, Japan; 3 Department of Medicine, Division of Diabetes, Metabolism, and Endocrinology, Showa University School of Medicine, Tokyo, Japan

**Keywords:** Low-density lipoprotein (LDL) cholesterol, Proprotein convertase subtilisin/kexin type 9 (PCSK9), Lipoprotein(a), Oxidized LDL, Small, dense LDL

## Abstract

**Background:**

Proprotein convertase subtilisin/kexin type 9 (PCSK9) is a key regulator of serum low-density lipoprotein (LDL) cholesterol levels. Recently, PCSK9 has additionally been related to metabolic risk factors such as the levels of triglycerides, apolipoprotein B (apoB), insulin, and glucose, as well as body mass index. The purpose of this study was to investigate correlations between serum levels of PCSK9 and apoB-containing atherogenic lipoproteins in patients with coronary artery disease (CAD).

**Methods:**

Serum levels of PCSK9 and lipoprotein(a) [Lp(a)]; small, dense LDL; and oxidized LDL were measured in 101 patients with CAD who were not receiving lipid-lowering therapy.

**Results:**

Serum hetero-dimer PCSK9 levels were positively correlated with serum levels of Lp(a) (*r* = 0.195, *p* = 0.05); small, dense LDL (*r* = 0.336, *p* = 0.0006); and oxidized LDL (*r* = 0.268, *p* = 0.008). Multivariate regression analyses showed that serum hetero-dimer PCSK9 was a significant predictor of serum levels of Lp(a) (*β* = 0.235, *p* = 0.01); small, dense LDL (*β* = 0.143, *p* = 0.03); and oxidized LDL (*β* = 0.268, *p* = 0.008).

**Conclusions:**

Serum PCSK9 levels were positively correlated with serum levels of Lp(a); small, dense LDL; and oxidized LDL in patients with CAD. This suggests that the interaction between serum PCSK9 and apoB-containing lipoproteins plays a role in establishing the atherosclerotic status of patients.

**Trial registration:**

UMIN Clinical Trials Registry, UMIN ID: C000000311.

## Background

Proprotein convertase subtilisin/kexin type 9 (PCSK9) is a key regulator of serum low-density lipoprotein (LDL) cholesterol levels [1, 2]. PCSK9, which is secreted by the liver into the circulation, binds the hepatic LDL receptors (LDLRs), causing their subsequent degradation [3, 4]. Although the mechanism underlying PCSK9-mediated degradation of LDLR is extremely complex, it bind to LDLR subsequently targeting them for intracellular destruction within the hepatocyte, resulting in an increase in LDL cholesterol levels [5–7]. Therefore, PCSK9 antibodies represent attractive candidates for lowering LDL cholesterol levels.

Elevated levels of lipoprotein(a) [Lp(a)]; small, dense LDL; and oxidized LDL are recognized as risk factors for atherosclerotic cardiovascular disease (ASCVD) [8–11]. The levels of both small, dense LDL and oxidized LDL may be lowered by statin therapy [12, 13]; however, the availability of pharmacological agents for lowering Lp(a) levels is limited. Therefore, Lp(a) levels represent a residual risk factor for cardiovascular events in this statin era [14]. Although monoclonal antibodies against PCSK9 have been reported to lower Lp(a) levels [15], the mechanisms underlying this effect are poorly understood.

Several demographic and metabolic parameters appear to correlate with serum PCSK9 levels, including plasma LDL cholesterol, high-density lipoprotein (HDL) cholesterol, triglycerides, apolipoprotein B (apoB), insulin, glucose, smoking, and body mass index [16–18]. In addition, circulating PCSK9 strongly correlates with intermediate-density lipoprotein particles, suggesting a link between PCSK9 and triglyceride-rich lipoprotein metabolism [19]. However, to our knowledge, correlations between serum levels of PCSK9 and small, dense LDL or oxidized LDL have not been evaluated to date. In addition, few studies concerning the association of PCSK9 with Lp(a) have been reported. Therefore, the aim of this study was to investigate correlations between serum levels of PCSK9 and apoB-containing atherogenic lipoproteins such as Lp(a); small, dense LDL; and oxidized LDL in patients with coronary artery disease (CAD).

## Methods

### Patients and study design

The present study is a post-hoc analysis of the Treatment With Statin on Atheroma Regression Evaluated by Intravascular Ultrasound With Virtual Histology (TRUTH) study, which was a prospective, open-labeled, randomized, multicenter trial performed at 11 Japanese centers [20]. In brief, 164 patients with angina pectoris, who were not receiving any lipid-lowering therapy, were randomly treated with either 4 mg/day of pitavastatin or 20 mg/day of pravastatin.

The patients included in the TRUTH study were considered for the present study if an adequate serum volume, before statin treatment, was available in frozen samples from these patients; a total of 101 patients met this inclusion criterion.

This study was conducted in accordance with the Declaration of Helsinki and with the approval of the ethical committees of Yokohama Sakae Kyosai Hospital. Each patient enrolled in the present study provided written informed consent.

### Laboratory analysis

Serum levels of total cholesterol, LDL cholesterol, HDL cholesterol, and triglycerides were measured using standard enzymatic methods (AU2700; Beckman Coulter, CA, USA) and commercial enzymatic kits (Kyowa Medex, Tokyo, Japan). Serum levels of two forms of PCSK9, mature (hetero-dimer) and furin-cleaved, were measured at a central laboratory (BML, Kawagoe, Japan) using sandwich enzyme-linked immunosorbent assays (ELISAs) [21]. It has been reported that furin-cleaved PCSK9 has no activity to regulate LDLR and serum LDL cholesterol or less activity than mature PCSK9 [21]. Serum oxidized LDL levels were measured by an enzyme immunoassay [22]. Serum Lp(a) levels were measured by a latex agglutination turbidimetric immunoassay using the commercially available Lp(a)-LATEX (Sekisui Medical Co., Ltd., Tokyo) with an autoanalyzer (JCA-BM8040; JEOL Ltd., Tokyo). Serum small, dense LDL levels were measured by a homogeneous assay (Denka Seiken Co., Ltd., Tokyo) [23].

### Statistical analysis

Statistical analysis was performed using StatView, version 5.0 (SAS Institute, Cary, North Carolina). The results are expressed as means ± SD or median values (range). Univariate and multivariate linear regression analyses were performed to assess the correlations between serum levels of apoB-containing atherogenic lipoprotein and biochemical parameters, including nominal variables (gender, hypertension, diabetes mellitus, and smoking) and numerical variables (age, body mass index, total cholesterol, LDL cholesterol, triglycerides, HDL cholesterol, apoA1, apoB, hetero-dimer PCSK9, and furin-cleaved PCSK9). The variables with a *p* value < 0.1 on univariate analysis were entered into multivariate models. Statistical significance was set at *p* < 0.05.

## Results

The baseline characteristics of the subjects are listed in Table 1. Eighty-four patients were of male gender with a mean age of 67 ± 10 years. Forty-five of the patients were additionally diabetic.

**Table 1:** Baseline characteristics of subjects

Age (years)	67 ± 10
Male (%)	84 (83 %)
Body mass index (kg/m2)	24.3 ± 3.4
Hypertension (%)	66 (65 %)
Diabetes (%)	45 (45 %)
Family history of CAD (%)	10 (10 %)
Smoking (%)	23 (23 %)
Treated with ACE inhibitors or ARBs (%)	55 (54 %)
Treated with CCBs (%)	54 (53 %)
Treated with beta-blockers (%)	10 (10 %)

Data are expressed as the means ± SD or number (%)

*ACE* angiotensin-converting enzyme, *ARBs* angiotensin-receptor blockers, *CCBs* calcium channel blockers

Serum levels of lipid, PCSK9, and apoB-containing lipoproteins are shown in Table 2. The mean levels of LDL cholesterol and apoB were 129 ± 31 mg/dl and 103 ± 24 mg/dl, respectively. Serum hetero-dimer PCSK9 levels were 145 ± 60 ng/ml. Median levels of Lp(a) were 16 mg/dl, and mean levels of small, dense LDL and oxidized LDL were 25.8 ± 13.9 mg/dl and 11.8 ± 8.5 U/ml, respectively.

**Table 2:** Serum levels of lipids, PCSK9, and atherogenic lipoprotein

Total cholesterol (mg/dl)	202 ± 34
LDL cholesterol (mg/dl)	129 ± 31
Triglycerides (mg/dl)	114 (36–573)
HDL cholesterol (mg/dl)	47 ± 11
Apolipoprotein A1 (mg/dl)	118 ± 20
Apolipoprotein B (mg/dl)	103 ± 24
Hetero-dimer PCSK9 (ng/ml)	145 ± 60
Furin-cleaved PCSK9 (ng/ml)	47 ± 32
Lipoprotein(a) (mg/dl)	16 (1–47)
Small, dense LDL (mg/dl)	25.8 ± 13.9
Oxidized LDL (U/ml)	11.8 ± 8.5

Data are expressed as means ± SD or median (range)

*LDL* low-density lipoprotein, *HDL* high-density lipoprotein, *PCSK9* proprotein convertase subtilisin/kexin type 9

**Fig. 1:**
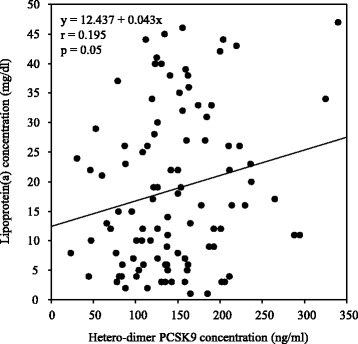
Correlation between serum levels of hetero-dimer PCSK9 and Lp(a). A significant positive correlation was observed between serum levels of hetero-dimer PCSK9 and Lp(a)

**Fig. 2:**
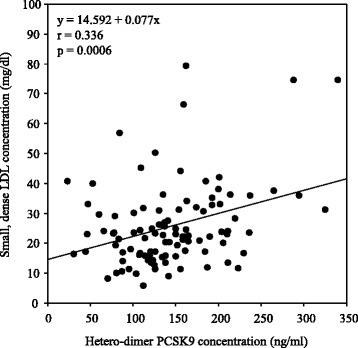
Correlation between serum levels of hetero-dimer PCSK9 and small, dense LDL. A significant positive correlation was observed between serum levels of hetero-dimer PCSK9 and small, dense LDL

**Fig. 3:**
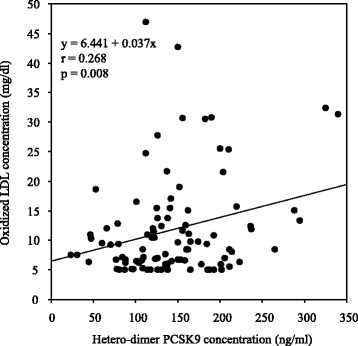
Correlation between serum levels of hetero-dimer PCSK9 and oxidized LDL. A significant positive correlation was observed between serum levels of hetero-dimer PCSK9 and oxidized LDL

Correlations between levels of apoB-containing lipoproteins and biochemical parameters are shown in Table 3. Univariate analysis indicated that total cholesterol, apoB, and hetero-dimer PCSK9 levels (Fig. 1) positively correlated with serum levels of Lp(a). Multivariate regression analysis showed that serum apoB and PCSK9 levels were significant positive predictors of Lp(a) levels. In addition, total cholesterol, LDL cholesterol, triglycerides, apoB, and hetero-dimer PCSK9 levels (Fig. 2) positively correlated with serum small, dense LDL levels. Multivariate regression analysis showed that apoB and hetero-dimer PCSK9 levels were significant positive predictors of small, dense LDL levels. Moreover, univariate and multivariate analyses showed that serum hetero-dimer PCSK9 levels positively correlated with serum oxidized LDL levels (Fig. 3). Therefore, weak, but significant, positive correlations were observed between serum levels of hetero-dimer PCSK9 and apoB-containing lipoproteins such as Lp(a); small, dense LDL; and oxidized LDL.

**Table 3:** Correlations between biochemical parameters and atherogenic lipoproteins levels

	Lipoprotein(a)				Small, dense LDL				Oxidized LDL			
	r	*p* value	β	*p* value	r	*p* value	β	*p* value	r	*p* value	β	*p* value
Age	0.129	0.2			−0.289	0.003	−0.056	0.41	−0.017	0.87		
Sex	−0.030	0.77			−0.072	0.48			0.084	0.42		
Body mass index	−0.180	0.08	−0.317	0.0009	0.233	0.02	0.028	0.68	−0.066	0.53		
Hypertension	0.060	0.55			−0.103	0.31			0.085	0.41		
Diabetes	−0.107	0.29			0.160	0.11			−0.100	0.33		
Smoking	−0.189	0.06	−0.273	0.004	−0.004	0.97			−0.073	0.48		
Total cholesterol	0.196	0.05	−0.241	0.24	0.711	<0.0001	0.209	0.19	0.080	0.44		
LDL cholesterol	0.144	0.15			0.611	<0.0001	−0.269	0.22	0.039	0.71		
Triglycerides	0.029	0.77			0.585	<0.0001	0.150	0.15	0.053	0.61		
HDL cholesterol	0.129	0.2			−0.109	0.28			0.069	0.5		
Apolipoprotein A1	0.056	0.58			0.022	0.82			0.047	0.65		
Apolipoprotein B	0.260	0.009	0.573	0.007	0.758	<0.0001	0.683	0.005	0.112	0.28		
Hetero-dimer PCSK9	0.195	0.05	0.235	0.01	0.336	0.0006	0.143	0.03	0.268	0.008	0.268	0.008
Furin-cleaved PCSK9	0.063	0.53			0.234	0.02			0.026	0.8		

Male gender, hypertension, diabetes, and smoking were assigned a value of 1. Female gender, normotension, non-diabetes, and non-smoking were assigned a value of 0

*LDL* low-density lipoprotein, *HDL* high-density lipoprotein, *PCSK9* proprotein convertase subtilisin/kexin type 9

## Discussion

The major findings of the present study are as follows: first, serum hetero-dimer PCSK9 levels were positively correlated with serum Lp(a) levels. Second, significant positive correlations were observed between serum levels of hetero-dimer PCSK9 and small, dense LDL or oxidized LDL. Finally, the serum hetero-dimer PCSK9 level was a significant predictor of serum levels of apoB-containing atherogenic lipoproteins.

Lp(a) is a LDL-like particle synthesized by the liver, that consists of an apoB100 molecule linked to a very large glycoprotein known as the apolipoprotein(a) [8, 24]. The biological role of Lp(a) is uncertain; however, elevated levels of Lp(a) have been identified as an independent risk factor for ASCVD [8, 9]. Several recent clinical trials have reported that PCSK9 antibodies represent promising novel candidate drugs for lowering the levels of both LDL cholesterol and Lp(a) [25–27]. However, the mechanism by which PCSK9 inhibitors reduce Lp(a) levels remains unclear. Serum Lp(a) levels in familial hypercholesterolemia with LDLR mutations have been shown to be elevated, suggesting that Lp(a) is catabolized via the LDLR pathway [28]; however, statins, whose main mechanism of action involves the upregulation of LDLR, are unable to reduce Lp(a) levels effectively [29]. In addition, Tada et al. recently reported that serum Lp(a) was elevated in patients with familial hypercholesterolemia caused by PCSK9 gain-of-function mutations to the same level as that in familial hypercholesterolemia caused by LDLR mutations [30]. This suggests that the LDLR plays an important role in Lp(a) catabolism. Furthermore, Romagnuolo et al. reported that Lp(a) catabolism is regulated by PCSK9 via LDLR in HEPG2 cells and primary human fibroblasts [31]. However, a previous study reported that apoB in Lp(a) does not interact with LDLR, suggesting that LDLR does not play a role in Lp(a) kinetics [24].

In order to understand the mechanism by which PCSK9 antibodies reduce Lp(a) levels, elucidation of the correlation between serum levels of PCSK9 and Lp(a) is required. A recent study by Nekaies et al. reported that plasma PCSK9 levels correlated positively with Lp(a) concentrations [32]. However, Yang et al. reported that plasma PCSK9 levels are not associated with Lp(a) levels [33]. Therefore, the correlation between plasma PCSK9 and Lp(a) levels remains controversial. Significant positive correlations were observed between serum levels of PCSK9 and Lp(a) in the present study; furthermore, serum apoB levels were additionally found to be positively correlated with serum Lp(a) levels. Our findings suggest that both PCSK9 and apoB interact with Lp(a). Variations in terms of correlation between PCSK9 and Lp(a) observed between patients may be attributed to differences in race, as both PCSK9 and Lp(a) levels vary considerably by race and other factors [18, 24, 34].

Small, dense LDL has been proposed to enhance atherogenicity owing to its higher rate of penetration into the arterial wall, prolonged plasma half-time, and lower affinity of LDLR [35]. Numerous previous studies have examined the association of small, dense LDL with traditional cardiovascular risk factors [36, 37]. In agreement with these previous reports, univariate analysis performed in the present study indicated that serum small, dense LDL levels were correlated with the concentration of total cholesterol, LDL cholesterol, triglycerides, and apoB. However, multivariate regression analysis showed that apoB and PCSK9 levels were significant predictors of small, dense LDL levels. Recently, Kwakernaak et al. found no association between plasma PCSK9 levels and small, dense LDL in healthy subjects [19]. However, Zhang et al. reported that plasma PCSK9 levels are positively associated with plasma small, dense LDL in patients with CAD; however, this association was not observed in subjects without CAD [38]. Therefore, the levels of plasma PCSK9 and small, dense LDL may be correlated in atherosclerotic patients. Although the exact mechanisms underlying the positive correlation between PCSK9 and small, dense LDL levels remain to be elucidated, PCSK9-induced LDLR degradation, which results in lower LDLR levels, potentially affects LDL subfractions, as characterized by the increased levels of small, dense LDL.

Oxidized LDL is also highly atherogenic and elevated levels of oxidized LDL recognized as a risk factor for ASCVD [11, 39]. Oxidized LDL levels have been reported to be positively correlated with PCSK9 concentration [40]. Consistent with this finding, we observed a positive correlation between serum levels of PCSK9 and oxidized LDL. This observation may be attributed to the involvement of PCSK9 in inflammatory and oxidative processes [41, 42]. In addition, inhibition of PCSK9 suppresses the inflammatory response induced by oxidized LDL in macrophages [43]. Further, circulatory PCSK9 is not only present in its free form, but is additionally complexed with apoB-containing lipoproteins [44]. Our findings indicate that serum PCSK9 levels positively correlated with apoB-containing lipoproteins such as Lp(a); small, dense LDL; and oxidized LDL.

Several recent clinical trials have reported that PCSK9 antibodies reduce Lp(a) levels [25–27]. However, there are no reports on the effects of PCSK9 antibodies on small, dense LDL or oxidized LDL. In view of the finding that PCSK9 antibodies reduced LDL cholesterol levels and particle numbers [45], we speculate that PCSK9 antibodies reduce small, dense LDL as well as oxidized LDL levels.

Plasma PCSK9 levels have been reported to be associated with severity of CAD as well as future risk of cardiovascular disease [46, 47]. In addition, plasma PCSK9 levels were significantly higher in patients with peripheral artery disease, especially those with extensive, severe, and complicated forms of the condition [40], suggesting that PCSK9 functions as a proatherogenic molecule [48]. Furthermore, PCSK9 is expressed in atherosclerotic plaques [41, 49]. Therefore, PCSK9-targeting strategies may represent potential therapeutic options for the treatment of ASCVD, whose mechanisms of action go beyond the reduction of LDL cholesterol.

The present study has several limitations in that it provides a post-hoc analysis of the results of the TRUTH trial. Plasma PCSK9 levels have been reported to be elevated in patients with acute myocardial infarction and associated with severity of CAD [46, 50]. In the present study, all subjects were patients with CAD; no control group was studied. In addition, serum PCSK9 levels were measured using frozen samples. Further, we did not evaluate the LDLR and PCSK9 expression in hepatocytes. Finally, the small number of patients included in the study resulted in low statistical power.

Despite these limitations, to the best of our knowledge, this is the first study to examine the correlations between serum levels of PCSK9 and apoB-containing atherogenic lipoproteins. A prospective study with a larger number of patients is required to confirm our conclusions.

## Conclusions

Serum PCSK9 levels were positively correlated with serum levels of Lp(a); small, dense LDL; and oxidized LDL in patients with CAD. This suggests that serum PCSK9 levels are correlated with those of apoB-containing lipoproteins in atherosclerotic patients.

## Abbreviations

apo: Apolipoprotein
ASCVD: Atherosclerotic cardiovascular disease
CAD: Coronary artery disease
HDL: High-density lipoprotein
LDL: Low-density lipoprotein
LDLR: LDL receptor
Lp(a): Lipoprotein(a)
PCSK9: Proprotein convertase subtilisin/kexin type 9
TRUTH: Treatment with statin on atheroma regression evaluated by intravascular ultrasound with virtual histology
